# CISD2 enhances the chemosensitivity of gastric cancer through the enhancement of 5‐FU‐induced apoptosis and the inhibition of autophagy by AKT/mTOR pathway

**DOI:** 10.1002/cam4.1169

**Published:** 2017-08-31

**Authors:** Yi Sun, Yingming Jiang, Jintuan Huang, Hao Chen, Yi Liao, Zuli Yang

**Affiliations:** ^1^ Department of Gastrointestinal Surgery The Sixth Affiliated Hospital (Guangdong Gastrointestinal and Anal Hospital) Sun Yat‐Sen University Guangzhou China; ^2^ Guangdong Provincial Key Laboratory of Colorectal and Pelvic Floor Diseases The Sixth Affiliated Hospital Sun Yat‐sen University Guangzhou China; ^3^ Guangdong Institute of Gastroenterology Guangzhou China

**Keywords:** 5‐FU, apoptosis, autophagy, chemotherapeutic sensitivity, cisd2, gastric cancer

## Abstract

Gastric cancer (GC) is a prevalent upper gastrointestinal tumor characterized by high morbidity and mortality due to imperfect screening systems and the rapid development of resistance to 5‐fluorouracil (5‐FU). CDGSH iron sulfur domain 2 (CISD2) has been recently regarded as a candidate oncogene in several types of tumors. It is, therefore, necessary to investigate its biological function and clinical significance in gastric cancer. In this study, the down‐regulated expression level of CISD2 in GC compared with adjacent normal tissues was evaluated by quantitative RT‐PCR and Western blotting. An immunohistochemical analysis indicated that CISD2 expression in GC was significantly correlated with age (*P *= 0.002), Lauren's classification (*P *= 0.001), and differentiation (*P *= 0.049). Two cell lines, MKN1 and BGC823, were used to analyze the role of CISD2 in gastric carcinogenesis and response to 5‐FU through CCK‐8 assays, the RT‐CES system, Transwell assays, flow cytometry, and confocal fluorescence microscopy. The overexpression of CISD2 resulted in reduced cellular growth and proliferation, inhibition of metastatic ability, and increased apoptosis. 5‐FU treatment increased endogenous as well as exogenous overexpression of CISD2 in GC cells. Further investigation revealed that CISD2 enhanced sensitivity to 5‐FU via an increase in apoptosis and inhibition of protective autophagy through the activation of the AKT/mTOR pathway. In conclusion, CISD2 is down‐regulated in gastric cancer, and its effects on the inhibition of cellular proliferation, metastatic ability, and increased chemotherapy sensitivity are mediated by antagonism to 5‐FU‐induced autophagy through the AKT/mTOR pathway.

## Introduction

Gastric cancer (GC) is one of the most prevalent malignant neoplasms worldwide, as 754,000 patients died from gastric cancer in 2015 according to WHO, which means that GC ranks fourth with respect to cancer‐related deaths 1. Approximately 43% of the total cases occur in China, which will also have approximately 480,000 new cases and 340,000 mortalities in 2015; GC is, therefore, the most common cause of cancer‐associated mortality 2. At present, most patients are diagnosed at an advanced stage due to a lack of typical clinical symptoms and imperfect screening systems, and thus surgical resection remains the only curative treatment for advanced‐stage GC. In addition, the high rates of recurrence and distant metastasis are the main reasons for poor survival, and therefore, it is essential to establish effective biologic adjuvant treatment for GC patients. Among the many types of drugs that are used to treat GC, 5‐fluorouracil (5‐FU) and 5‐FU‐based chemotherapy regimens are the mainstream treatments that have been widely used up to now 3, 4. Due to the rapid development of 5‐FU resistance and that high doses of drugs will result in several side effects on healthy cells, new adjuvant drugs such as Oxaliplatin, Paclitaxel, and Docetaxel combined with 5‐FU have led to an increased response rate in advanced GC 5, 6, 7. The identification of new therapeutic strategies and more sensitive screening systems toward patient subpopulations who are most likely to respond to chemotherapy will be the keys to improve the curative effect of chemotherapy and the prognosis of gastric cancer.

CDGSH iron sulfur domain 2 (CISD2)was originally regarded as a survival gene based on its roles in the maintenance of the integrity and function of mitochondria 8, calcium metabolism 9, redox reaction 10, and longevity 11, 12. Since tumor cells have the capacity for unlimited growth and show a long lifespan, researchers have gradually focused on the role of CISD2 in tumors. Recent studies have shown that CISD2 plays an important role in the tumorigenesis and progression of different tumors. In human epithelial breast cancer and early‐stage cervical cancer, CISD2 was found to be central to breast cancer proliferation 13, and correlate with pelvic lymph node metastasis and prognosis 14, relatively. In hepatocellular carcinoma, high CISD2 expression is associated with poor patient prognosis 15. In addition, CISD2 is involved in spinal cord injuries and serves as a neuroinflammatory suppressor in glial cells 16. However, the profound biological functions and molecular mechanisms of CISD2 in different tumors may vary and are still not well defined. Thus, further studies on the precise roles of CISD2 in human gastric cancer and the clarification of its exact mechanism will help to determine the potential medical applications. In this manuscript, we focus on the roles of CISD2 in tumor development, metastasis, and especially its role in the reaction to chemotherapeutic drugs in gastric cancer.

Here, we have investigated the expression of CISD2 in tumor samples from a large cohort of gastric cancer patients and GC cell lines. An enhancement in the expression of CISD2 in GC cells was significantly associated with a reduction in cell proliferation and tumor growth, inhibition of invasion and migration abilities, enhancement in the chemotherapeutic sensitivity to 5‐FU, and antagonism to 5‐FU‐induced autophagy through activation of the AKT/mTOR pathway in GC cells. Taken together, our findings provide new concepts that can be used to enhance the validity of 5‐FU in gastric cancer. Our results suggest that CISD2 plays an important role in human gastric cancer cells and may be a promising chemotherapeutic target for cancer treatment.

## Materials and Methods

### Patients and tissue samples

This study consisted of 52 GC sets of fresh‐frozen tissue samples that originated from primary gastric cancer and adjacent normal tissue obtained from patients at the Sixth Affiliated Hospital of Sun Yat‐sen University in 2015. 197 pairs of GC and adjacent normal tissue samples were obtained from the tumor bank of the Department of Pathology of Sun Yat‐Sen University. The patients, who underwent surgery without chemotherapy or radiotherapy between January 2007 and December 2008, were followed up until May 2014 to collect pathology reports and conditions. This study was conducted in compliance with the Code of Ethics of the World Medical Association and was approved by the research ethics committee of Sun Yat‐Sen University, China. Written Informed consent was obtained from all individual patients in this study.

### Tissue microarray and immunohistochemistry

Tissue microarrays (TMAs) were constructed and immunohistochemistry (IHC) was performed on the TMA slides as previously described 17. Each sample's histological diagnosis was made according to the World Health Organization (WHO) criteria and the AJCC/UICC TNM classification system 18. Briefly, the slides were incubated with a CISD2 human polyclonal antibody (diluted 1:800, Proteintech, Chicago, USA, 13318‐1‐AP). The antibody diluent buffer was used as a negative control. After the slides were stained with DAB and counterstained with hematoxylin, CISD2 staining was examined by Image‐Pro‐Plus (version 6.0, Media Cybernetics, Rockville, USA) as previously described 19. Receiver operating characteristic curve analysis was performed to select cut‐off scores (6.45). Representative fields were captured by a Leica DM4000B inverted microscope (Leica, Wetzlar, Germany).

### Cell culture and treatment

Ten human gastric cancer cell lines (AGS, SGC7901, BGC823, MKN1, MKN28, MKN45, VCC2, SNU1, KATO3, and HGC27) and an immortalized gastric mucosal cell line (GES‐1) were obtained from the cell line bank of the Chinese Academy of Sciences (Shanghai, China). All were authenticated and confirmed to be mycoplasma‐free. These cells were cultured in the recommended medium supplemented with 10% fetal bovine serum (HyClone, South Logan, USA) and 1% penicillin‐streptomycin at 37°C in a humidified atmosphere containing 5% CO_2_ and 95% air; and the medium was changed every 2 days. Cells in the logarithmic phase of growth were used for further experiments. For treatment, the cells were treated with 5‐FU or/and the autophagy inhibitor 3‐Methyladenine (3‐MA) (Sigma, St. Louis, USA). 5‐FU and 3‐MA were diluted in dimethyl sulfoxide and bidistilled water, respectively, and were stored at −20°C before use. The cells were divided into six groups as follows: control, CISD2, control+3‐MA, control+5‐FU, CISD2+5‐FU, and control+3‐MA+5‐FU. Following 24 h of seeding, the medium was replaced with fresh medium containing no compounds (control group), 5‐FU with or without 3‐MA for an additional 48 h. In the case of 3‐MA treatment, the cells were pretreated for 2 h with 5 mmol/L 3‐MA before 5‐FU treatment.

### Establishment of stably transfected cell lines

The *CISD2* construct was generated by subcloning PCR‐amplified full‐length human *CISD2* cDNA into pCDH‐CMV‐MCS‐EF1‐copGFP and pCDH‐CMV‐MCS‐EF1‐puro (System Biosciences, Palo Alto, USA). HEK‐293T cells were cotransfected with the resultant lentiviral recombinant vector or empty vector along with packaging plasmids (pMD2.G and psPAX2) (Addgene, Cambridge, USA) according to the manufacturer's instructions; the lentiviral supernatants were used to infect target cells. MKN1 and BGC823 cells, both of which have a low level of endogenous CISD2 expression, were transfected with lentivirus encoding CISD2 overexpression or the control using Lipofectamine3000 (Invitrogen, Carlsbad, USA) according to the manufacturer's protocols. The transfection of MKN1 and BGC823 cells with GFP fluorescence was confirmed by flow cytometry, and the antibiotic‐resistant transfected MKN1 and BGC823 cells were selected with 1.0 and 2.0 *μ*g/mL puromycin (Invitrogen, Carlsbad, USA), respectively. Additionally, in order to assess the induction of autophagy, the cells with stable ectopic expression of CISD2 with antibiotic‐resistance and the corresponding control cells were secondarily transfected with lentivirus encoding mRFP‐LC3‐GFP according to the standard protocols mentioned above. All transfections were confirmed by Western blotting and fluorescence microscopy.

### RNA isolation and quantitative real‐time polymerase chain reaction

Total RNA isolation, reverse transcription and quantitative real‐time polymerase chain reaction (qRT‐PCR) were performed as previously described 17. The primers used are listed in Table 1. The relative RNA levels in each sample were determined by standard curves. *β*‐actin served as an internal control for the cDNA input in the qRT‐PCR assay.

**Table 1:** Primers used for qRT‐PCR

Name	Primer sequence forward	Primer sequence reverse
CISD2 *β*‐actin	5′‐GTGGCCCGTATCGTGAAGG‐3′ 5′‐CAATGAGCTGCGTGTGGCT‐3′	5′‐CTAGCGAACCCGGTAATGCTT‐3′ 5′‐TAGCACAGCCTGGATAGCAA‐3′

### Western blotting

Total proteins were extracted from transfected GC cells. Protein samples were separated by 10–12% sodium dodecyl sulfate‐ polyacrylamide gel electrophoresis (SDS‐PAGE) and transferred to a polyvinylidene fluoride (PVDF) membrane (Pall, New York, USA). After blocking with 5% skim milk for 1 h, the proteins were detected using specific antibodies against the following: human CISD2 (diluted 1:1000, Proteintech, Chicago, USA, 13318‐1‐AP), Beclin1, LC3A/B, ATG3, ATG5, ATG7 (diluted 1:1000, Cell Signaling Technology, Danvers, USA, Autophagy Antibody Sampler Kit #4445), total and cleaved poly ADP‐ribose polymerase (PARP), Caspase‐3, Caspase‐9 (diluted 1:800‐1:1000, Cell Signaling Technology, Danvers, USA, Apoptosis Antibody Sampler Kit #9915), BCL‐2, BCL‐xl, BAD, total and phosphorylated AKT/mTOR (diluted 1:1000, Cell Signaling Technology, Danvers, USA), and GAPDH (diluted 1:10,000, Proteintech, Chicago, USA) respectively. After the membranes were incubated with the primary antibodies overnight at 4°C and HRP‐conjugated secondary antibodies (dilution 1:5000, Santa Cruz, Dallas, USA) for 1 h at room temperature, the proteins were then detected with enhanced chemiluminescence reagent and were observed by the Bio‐Rad ChemiDoc^®^ Touch Imaging System (Bio‐Rad, Hercules, USA). The density of each band was quantified by scanning densitometry and was analyzed by ImageJ software (NIH, Bethesda, USA). The corresponding figure shows a representative blot from the three experiments, which had similar results, and all protein expression levels were evaluated relative to GAPDH expression.

### Cell proliferation and IC50 assay

Cells were seeded into 96‐well plates at a density of 1 × 10^4^ cells per well for the Cell Counting Kit‐8 (CCK‐8) assay (Beyotime, Shanghai, China), and cell viability was assessed in a microplate reader (Thermo Fisher, Waltham, USA) after 0–6 days of incubation. For the half maximal inhibitory concentration (IC50) assay, cells were treated with 5‐FU at concentrations that ranged from 1 to 4112 *μ*mol/L for 72 h. The IC50 concentration was evaluated based on concentration‐response curves by nonlinear regression analysis using GraphPad Prism5 software (San Diego, USA). All data are shown as the mean ± standard deviation (SD) for at least three independent experiments, and each experiment was performed in six parallel wells.

### Real‐time continuous assessment of cellular growth

The RT‐CES microelectronic cell sensor system (ACEA Biosciences Inc., San Diego, USA) was used to confirm the number of living cells and cellular growth. The real‐time electronic sensor provided a continuous and quantitative measurement of the cell index in each well 20. According to the manufacturer's instructions, cells were seeded at a density of 5 × 10^3^ cells per well and were incubated in the RT‐CES system incubator for 7 days. Data were automatically collected every 15 min by the analyzer. This experiment was performed at least in triplicate, and each experiment was performed in four parallel wells.

### Clonogenic assay

Cells were plated on 6‐well plates (700 cells/well) with or without 5‐FU treatment and cultured for 2 weeks. After fixation in 4% paraformaldehyde for 20 min at room temperature, the colonies were stained with 1% crystal violet. The number of colonies that consisted of more than 50 cells was counted by ImageJ software. The experiment was performed at least three times.

### Invasion, migration, and would healing assays

The invasion and migration assays were performed as previously described 17. Briefly, cells were seeded at a density of 5 × 10^5^ in the upper chambers. After fixation, the cells were stained with 4′,6‐diamidino‐2‐phenylindole (DAPI) and were counted under an inverted microscope. For the wound healing assay, cells were seeded into 6‐well plates and cultured to near confluence. Then, a sterile pipette tip was used to generate a scratch. After incubation for 0–6 days, the cells were photographed under a bright‐field microscope. The experiments were performed at least in triplicate, and each experiment was performed in three parallel wells.

### Cell cycle and apoptosis assay

The effect of CISD2 on the cell cycle distribution and apoptosis of GC cells was examined by flow cytometry as previously described 21. Briefly, the cells were seeded in 6‐well plates at a density of 1 × 10^5^ cells per well and were harvested after treatment with 50 *μ*mol/L 5‐FU for MKN1 cells and 500 *μ*mol/L for BGC823 cells. For cell cycle assay, cells were sorted by FACS Calibur (BD Biosciences, Franklin Lakes, USA), and the profiles were analyzed by ModFit software (version 3.0, Verity Software House, Topsham, USA). The cell apoptosis assay was performed by dual staining with Annexin V‐Phycoerythrin (PE) and 7‐amino‐actinomycin D (7‐AAD). The Annexin V‐positive cells were counted as apoptotic cells, and the data were analyzed using FlowJo software (version 7.6.1, TreeStar, Oregon, USA). The experiments were performed at least in triplicate.

### Confocal fluorescence microscopy

Confocal microscopic analysis was performed to examine the cellular autophagy level and the mechanisms of 5‐FU‐induced autophagy in cells stably transfected with mRFP‐LC3‐GFP lentivirus. Briefly, cells were seeded onto 6‐well chamber slides at 30% confluence, with or without 5 mmol/L of 3‐MA for 2 h, and were then cotreated with 5‐FU for 48 h. After an incubation for 48 h, the cells reached 60–80% confluence and were washed with PBS buffer, fixed in 4% paraformaldehyde, counterstained with DAPI and mounted with Fluorosave. The LC3 localization and autophagosome formation were analyzed by a TCS SP2 laser scanning confocal microscope (Leica, Wetzlar, Germany) using a standard fluorescein channel for imaging the autophagic signal. The number of punctate autophagic vacuoles was measured by ImageJ software. This experiment was performed at least in triplicate, and each measurement was performed in ten visual fields.

### Statistical analyses

Statistical Product and Service Solutions (SPSS, version 20.0, IBM, New York, USA) was used for all statistical analyses. Continuous variables were expressed as the mean ± SD and were analyzed by Student's *t*‐test. The differences in categorical variables were analyzed using the Chi‐square test or Fisher's exact test. Survival analyses were plotted using the Kaplan–Meier method and were compared using the log‐rank test. Differences at *P *<* *0.05*,* which was derived from two‐tailed tests, were considered statistically significant.

## Results

### Expression status of CISD2 in human GC tissues and cell lines

**Figure 1:**
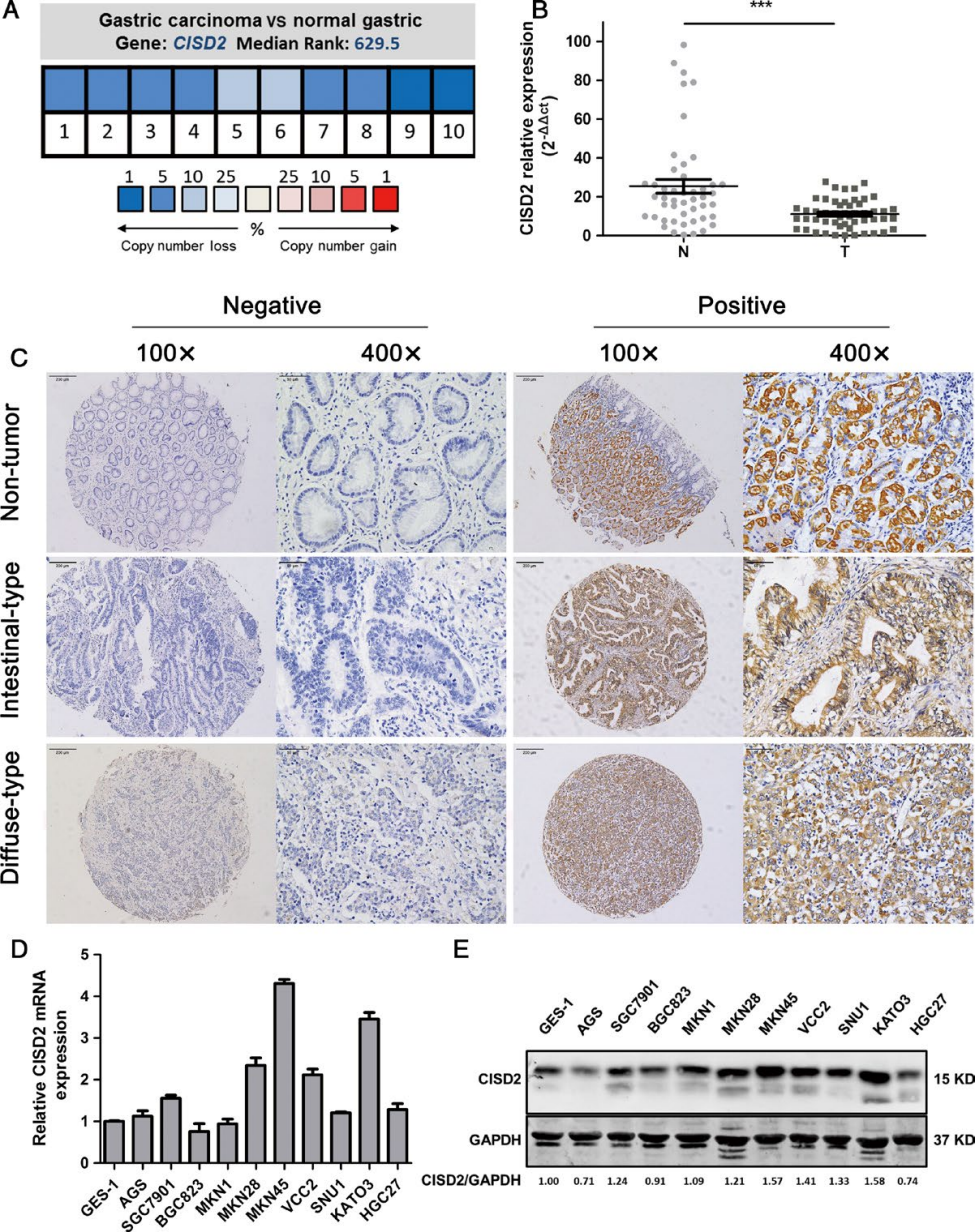
Expression status of CISD2 in human GC tissues and cell lines. (A) *CISD2* DNA copy number alterations in samples from gastric tumor and normal tissue were analyzed by the Oncomine microarray database. Pooling of 10 analyses from TCGA Gastric and Deng Gastric microarray studies shows a frequent copy number loss of *CISD2* in human gastric cancer compared with normal tissues. (*P *<* *0.001*)* (B) The expression of *CISD2* mRNA levels in 52 pairs of gastric cancer tissues and adjacent normal tissues was measured by qRT‐PCR. The data are expressed as 2^−ΔΔct^. (C) The expression of CISD2 protein was examined by IHC staining in nontumor gastric tissue, intestinal‐type gastric cancer and diffuse‐type gastric cancer. Representative IHC images of CISD2 are shown. Original magnification: 100× and 400×. (D and E) CISD2 mRNA and protein levels in gastric cancer cell lines were analyzed by Western blotting and qRT‐PCR. Data are shown as mean ± SD of three independent experiments. (*P <* *0.05, **P <* *0.005, ***P <* *0.001)

Through an analysis of *CISD2* DNA copy number alterations in the Oncomine microarray database, which contains data from gastric cancer patients, a frequent copy number loss of *CISD2* was observed in human GC compared with normal gastric tissues (Fig. 1A). Moreover, the expression of *CISD2* mRNA levels in an independent set of 52 pairs of GC tissues were evaluated by qRT‐PCR and compared with corresponding adjacent normal tissues, it was found that the mRNA expression levels of *CISD2* were down‐regulated in primary GC tissues (11.09 ± 1.027 vs. 25.52 ± 3.531, *P *<* *0.0001) (Fig. 1B). When CISD2 protein expression was examined in 197 pairs of paraffin‐embedded human GC and adjacent normal tissues by IHC, the CISD2 protein was found to be localized in the cytoplasm, and the IHC staining indicated more GC tissues with negative or lower CISD2 expression than the corresponding paired normal tissues (Fig. 1C, Table 2). Among 197 paired normal tissues, weak expression of CISD2 was detected in 56 cases, while high or moderate expression was detected in 141 cases. Among tissues from gastric cancer patients, negative or weak expression of CISD2 protein was found in 123 cases, while 74 cases showed high or moderate expression. The percentage of gastric tumor tissues with a low level of CISD2 protein expression was much greater than that of the matched normal tissues (61.92% vs. 28.42%, *P *<* *0.001). Furthermore, mRNA and protein levels of CISD2 were also measured in ten GC cell lines by qRT‐PCR and Western blotting respectively (Fig. 1D and E). Compared with the immortalized gastric mucosal cell line GES‐1, CISD2 expression was relatively low in the eight GC cell lines examined, especially in MKN1 and BGC823, while CISD2 expression was relatively high in MKN45 and KATO3 cells. Based on these results, these two cell lines were selected for further analysis. Together, these data clearly indicate that CISD2 is down‐regulated in GC tissues.

**Table 2:** Comparison of CISD2 expression in gastric cancer tissue and paired noncancerous tissue by IHC (*n *= 197)

	Cases	CISD2 expression		*χ* ^2^	*P* value
		Negative or weak	Positive or high		
Gastric cancer tissue	197	122 (61.92%)	75 (38.07%)	44.639	<0.001
Paired normal tissue	197	56 (28.42%)	141 (71.57%)		

### Association of CISD2 protein expression with the clinicopathological characteristics of GC patients

Consistently, the association between CISD2 expression and the clinicopathological characteristics of GC was assessed in 197 patients with primary GC. The statistical analysis showed that the presence of the CISD2 protein was significantly correlated with the clinicopathological parameters. As shown in Table 3, the presence of CISD2 protein was elevated in patients aged >=60 (*P *= 0.002), in those with the intestinal type of gastric cancer (*P *= 0.01), and in those with well‐differentiated cancers (*P *= 0.049). No significant differences were found with respect to gender, the WHO histological type and TNM classification, vessel invasion or perineural invasion. However, a clear trend toward an increase in N stage with a decrease in positive CISD2 expression was detected. In this study, the correlation of CISD2 protein expression and the postoperative survival of GC patients was also evaluated. However, no significant correlations were found (data not shown).

**Table 3:** Association between CISD2 expression and clinicopathological characteristics of GC patients (*n *= 197)

Indicator	*n *= 197	CISD2 expression, *n* (%)		*χ* ^2^	*P* value
		Negative(122)	Positive(75)		
Gender				0.821	0.365
Male	137	82 (59.9%)	55 (40.1%)		
Female	60	40 (66.7%)	20 (33.3%)		
Age				9.609	0.002
<60 years	88	65 (73.9%)	23 (26.1%)		
>=60 years	109	57 (52.3%)	52 (47.7%)		
Histologic type				2.679	0.444
Tubular or papillary adenocarcinoma	162	99 (61.1%)	63 (38.9%)		
Mucinous adenocarcinoma	9	5 (55.6%)	4 (44.4%)		
Signet‐ring‐cell carcinoma	23	17 (73.9%)	6 (26.1%)		
Others	3	1 (33.3%)	2 (66.7%)		
Lauren's classification					
Diffuse type	96	72 (75%)	24 (46.2%)	15.201	0.001
Intestinal types	78	36 (46.2%)	42 (53.8%)		
Mixed type	23	14 (61.9%)	9 (39.1%)		
Differentiation grade				6.043	0.049
Well	25	10 (40.0%)	15 (60.0%)		
Moderately	23	14 (60.9%)	9 (39.1%)		
Poorly	149	98 (65.8%)	51 (34.2%)		
T stage				1.485	0.847
T1	18	12 (66.7%)	6 (33.3%)		
T2	26	16 (61.5%)	10 (38.5%)		
T3	103	66 (64.1%)	37 (35.9%)		
T4	50	28 (56.0%)	22 (44.0%)		
N stage				4.703	0.195
N0	53	28 (52.8%)	25 (47.2%)		
N1	65	39 (60.0%)	26 (40.0%)		
N2	57	38 (66.7%)	19 (33.3%)		
N3	22	17 (77.3%)	5 (22.7%)		
M stage				0	0.987
M0	168	104 (61.9%)	64 (38.1%)		
M1	29	18 (62.1%)	11 (37.9%)		
TNM stage				3.841	0.481
I	28	19 (67.8%)	9 (32.1%)		
II	33	16 (48.5%)	17 (51.5%)		
III	67	43 (64.2%)	24 (36.2%)		
IV	69	44 (63.8%)	25 (38.1%)		
Vessel invasion				0.782	0.458
No	113	67 (59.3%)	46 (40.7%)		
Yes	84	55 (65.5%)	29 (34.5%)		
Perineural invasion				0.369	0.560
No	97	58 (59.8%)	39 (40.2%)		
Yes	100	64 (64.0%)	36 (36.0%)		

### Up‐regulation of CISD2 inhibits cell proliferation and the tumor‐promoting activity of GC cells

**Figure 2:**
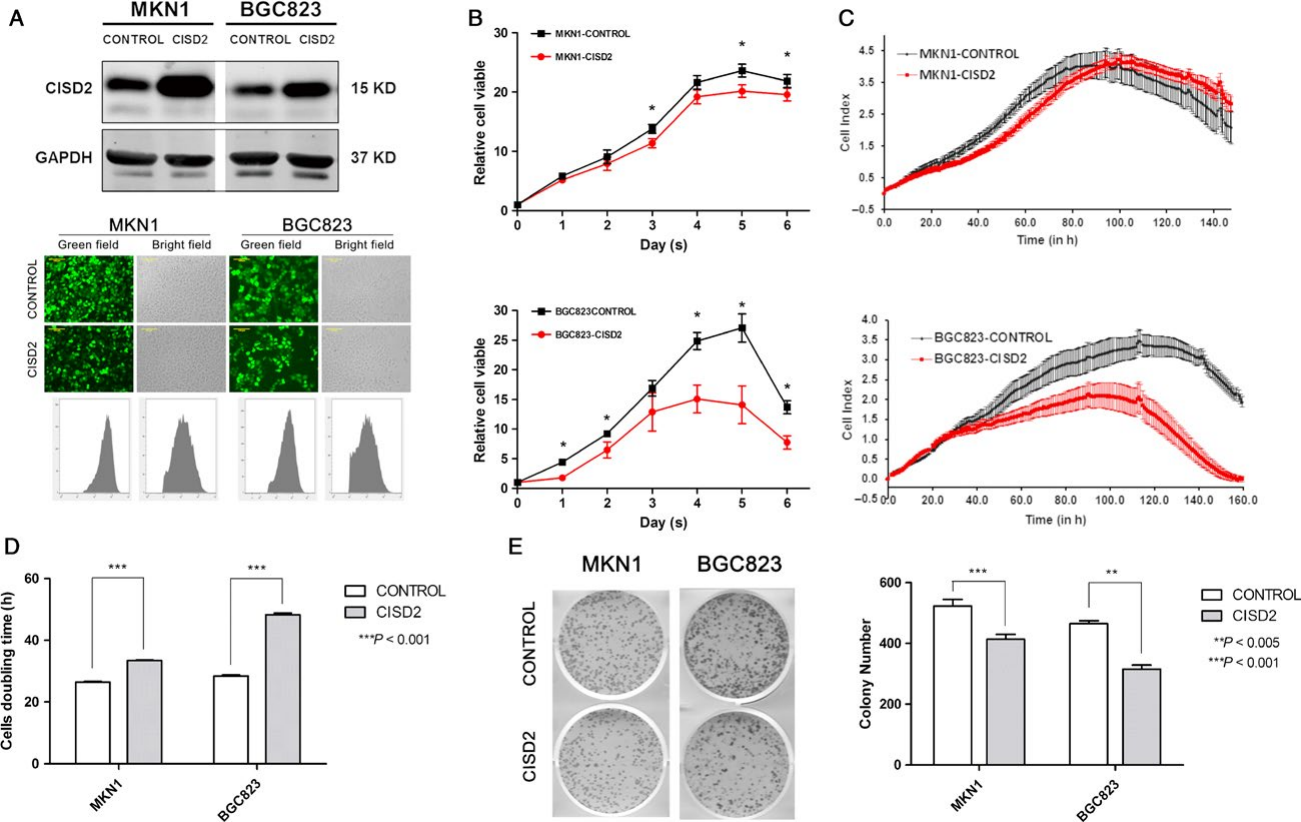
Up‐regulation of CISD2 inhibits cell proliferation and the tumor‐promoting activity of GC cells. (A) Western blotting analysis of the efficacy of CISD2 overexpression in MKN1 and BGC823 cells transfected with lentivirus. Bright‐field images and the corresponding fluorescence images are shown in the middle panel, and flow cytometric analysis images are shown in the bottom panel. (B) Cell viability was evaluated by CCK‐8 assay at 0–6 days after seeded (*n* = 6). (C and D) The growth curves and doubling time of CISD2 overexpression and control cells were monitored by RT‐CES biosensor. Data are from four replicates (shaded areas are SEM). (E) The colony formation assay of CISD2 overexpression and control cells (left panel), and colony numbers of each group analysis was performed as mean ± SD of three independent experiments (right panel). (*P <* *0.05, **P <* *0.005, ***P <* *0.001)

For further evaluation of the potential role of CISD2 in GC cell proliferation and carcinogenesis, cell lines that stably overexpressed CISD2 were established. The CISD2 protein expression levels in both transfected and control cells were determined by Western blotting, which showed a dramatic increase in expression in MKN1 and BGC823 cells compared with the empty vector‐transfected cells (Fig. 2A top panel). The transfection efficiency of these two cell lines, which was further confirmed by flow cytometry, was 80.92% and 69.78%, respectively (Fig. 2A bottom panel). Cell viability was assessed by CCK‐8 assay, and it was found that the number of MKN1 and BGC823 cells in which CISD2 was overexpressed was decreased significantly (both *P *<* *0.05 vs. control), which illustrated CISD2 overexpression reduced cellular growth and proliferation compared with the empty vector‐transfected control cells (Fig. 2B). Then, the dynamic changes in cell growth were measured by the by RT‐CES system, which is a new, real‐time biosensor that monitors cellular growth based on impedance 22. Two cell lines with CISD2 overexpression had demonstrated a reduction in growth rates compared with the control cells, and in BGC823 cell this reduction was more pronounced when the cells were approaching confluence (Fig. 2C). MKN1 cells (33.42 ± 0.20 h vs. 26.48 ± 0.17 h, *n* = 4) and BGC823 cells (48.28 ± 0.52 h vs. 28.41 ± 0.39 h, *n* = 4) that overexpressed CISD2 had a longer doubling time (both *P *<* *0.001 vs. control) (Fig. 2D). A colony formation assay revealed that MKN1 cells (414 ± 13.07 vs. 523 ± 17.57, *P *= 0.0009) and BGC823 cells (315 ± 11.22 vs. 465 ± 7.85, *P *= 0.0048) that overexpressed CISD2 formed fewer colonies than the control cells (Fig. 2E). Taken together, these results suggest that the up‐regulation of CISD2 could restrain cell proliferation and carcinogenesis of GC cells.

### CISD2 inhibits the invasion and migration abilities of GC cells

**Figure 3:**
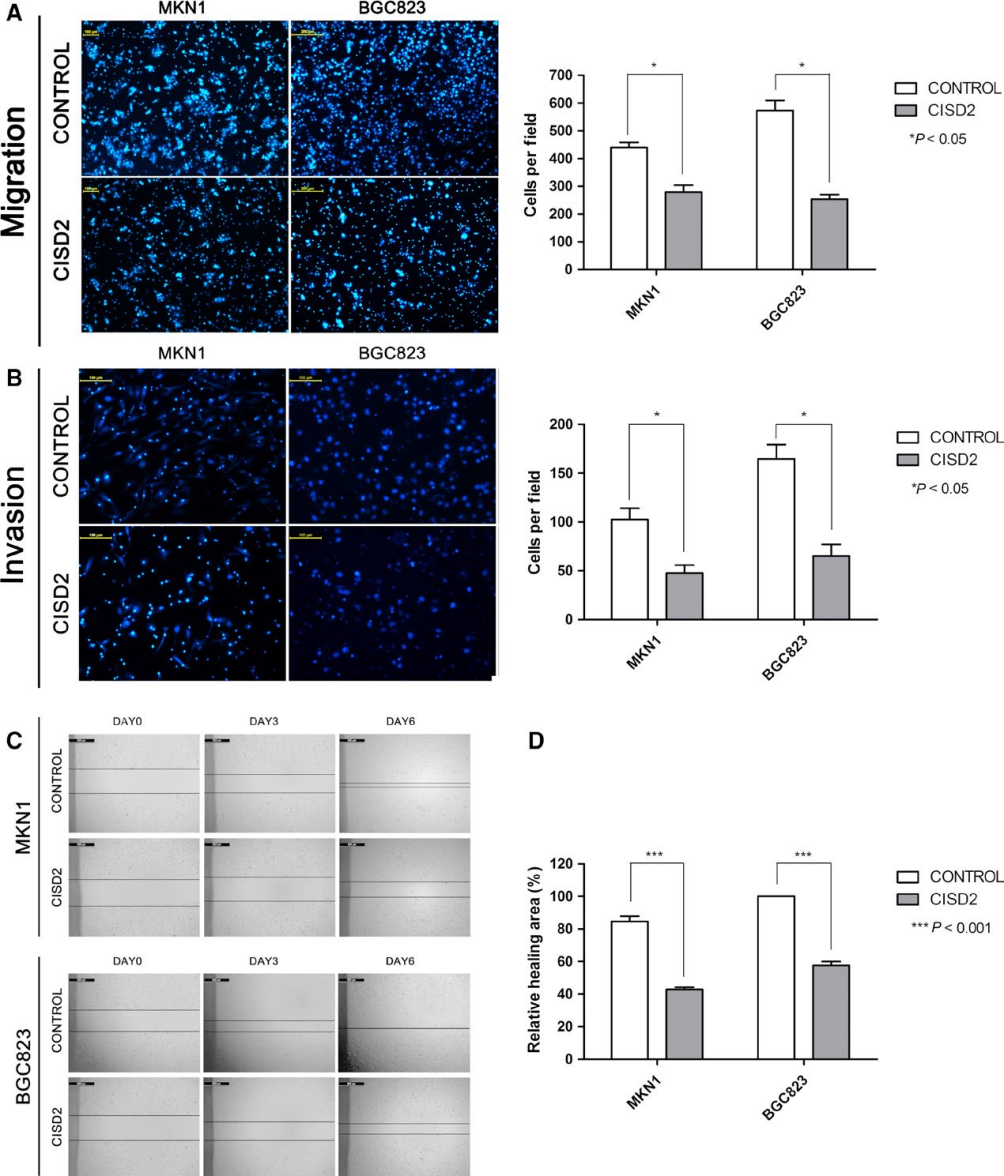
CISD2 inhibits the invasion and migration abilities of GC cells. (A and B) The migration and invasion of MKN1 and BGC823 cells were evaluated by Transwell assays. Original magnification: 100×. Scale bars: 100* μ*mol/L (left panel). The data are presented as the mean ± SD of three independent experiments (right panel). (C,D) Representative micrographs of the wound healing assay on the 0, 3rd, 6th day in MKN1 and BGC823 cells. The relative healing areas were presented as the mean ± SD of three independent experiments. (*P <* *0.05, **P <* *0.005, ***P <* *0.001)

To determine the effect of CISD2 on the migration and invasiveness of GC cells, Transwell migration, Matrigel invasion, and wound healing assays were performed. The number of migrated MKN1 cells in the CISD2 group was significantly decreased compared with that in the control group (278.33 ± 20.95 vs. 439.33 ± 14.70*, P *= 0.0232); the same result was observed in BGC823 cells in the CISD2 group (252.67 ± 13.87 vs. 339.67 ± 22.45, *P *= 0.0058) (Fig. 3A). Additionally, CISD2 remarkably inhibited cell invasion abilities of MKN1 ‐(47.67 ± 6.55 vs. 102.33 ± 9.53, *P *= 0.0401) and BGC823 cells (65.00 ± 9.90 vs. 164.67 ± 11.84*, P *= 0.0229) (Fig. 3B). In the wound healing assay, cells in the control group showed near closure of the wound on the 6th day after the scratch was generated, whereas cells in the CISD2 group were unable to heal the wound (Fig. 3C). The healed areas in the CISD2 group were fewer in number than those of the control group for MKN1 (42.73% ± 1.23% vs. 84.64% ± 2.61%*, P *= 0.0006) and BGC823 cells (57.67% ± 1.89% vs. 100%, *P *= 0.0010) (Fig. 3D). These results indicated that the up‐regulation of CISD2 could inhibit the migration and invasion abilities of GC cells.

### CISD2 is associated with sensitivity of gastric cancer cells to 5‐FU

**Figure 4:**
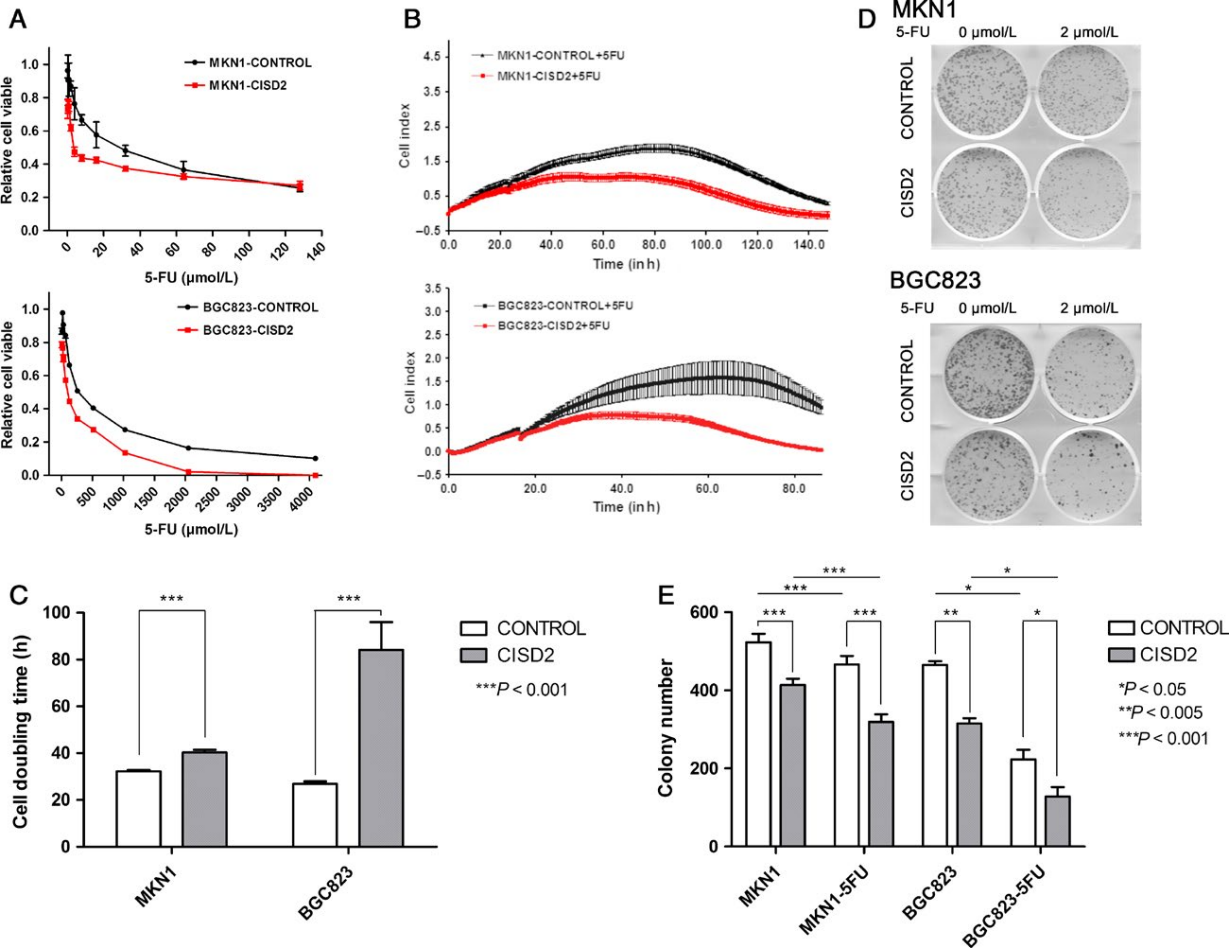
CISD2 is associated with sensitivity of gastric cancer cells to 5‐FU. (A) The IC50 curve of CISD2 overexpression and control cells were evaluated by CCK‐8 assay at 72 h after 5‐FU treatment (*n* = 6). (B,C) The growth curves and the doubling time of CISD2 overexpression and control cells exposed to 5‐FU were obtained by RT‐CES biosensor. Data are from four replicates (shaded areas are SEM). (D and E) The colony formation assay of CISD2 overexpression and control cells under 5‐FU treatment in MKN1 and BGC823 cells (2 *μ*mol/L and 5 *μ*mol/L, respectively) for continuous 2 weeks, and the colony numbers of each group performed as mean ± SD of three independent experiments. (*P <* *0.05, **P <* *0.005, ***P <* *0.001)

Based on the CCK‐8 assay, ectopic CISD2 expression significantly enhanced the sensitivity of MKN1 and BGC823 cells to 5‐FU and was important in the reduction in their IC50 values. As shown in Fig. 4A, CISD2 dramatically reduced the IC50 on average in MKN1 (7.80 *μ*mol/L ± 2.11 *μ*mol/L vs. 26.42 *μ*mol/L ± 3.95 *μ*mol/L, *P *= 0.0063) and BGC823 cells (91.62 *μ*mol/L ± 9.27 *μ*mol/L vs. 314.10 *μ*mol/L ± 27.4 *μ*mol/L, *P *= 0.015) compared with the control cells. At the same time, the RT‐CES system was used to examine the sensitivity of cells in the CISD2 and control groups to 5‐FU. The cell growth rates in the CISD2 group were decreased (Fig. 4B), and the doubling time was obviously increased in MKN1 (40.41 ± 1.15 h vs. 32.32 ± 0.58 h, *n* = 4), and BGC823 cells (84.19 ± 11.85 h vs. 26.96 ± 0.95 h, *n* = 4) (both *P *<* *0.0001) compared with the control group in response to 5‐FU (Fig. 4C). When the colony formation ability of cells that overexpressed CISD2 was examined after exposure to 5‐FU, a significant inhibition was observed in clonality after 5‐FU treatment in MKN1 and BGC823 cells (Fig. 4D). Consistently, the colony number in the CISD2 group was less than that in the control group after 5‐FU treatment in MKN1 (319.33 ± 15.92 vs. 466.67 ± 17.52, *P *<* *0.0001) and BGC823 cells (128 ± 19.95 vs. 233.34 ± 20.61, *P *= 0.0064) (Fig. 4E). These data show that CISD2 overexpression results in an increased sensitivity of MKN1 and BGC823 cells to 5‐FU.

### CISD2 is associated with 5‐FU‐induced apoptosis but not cell cycle arrest

**Figure 5:**
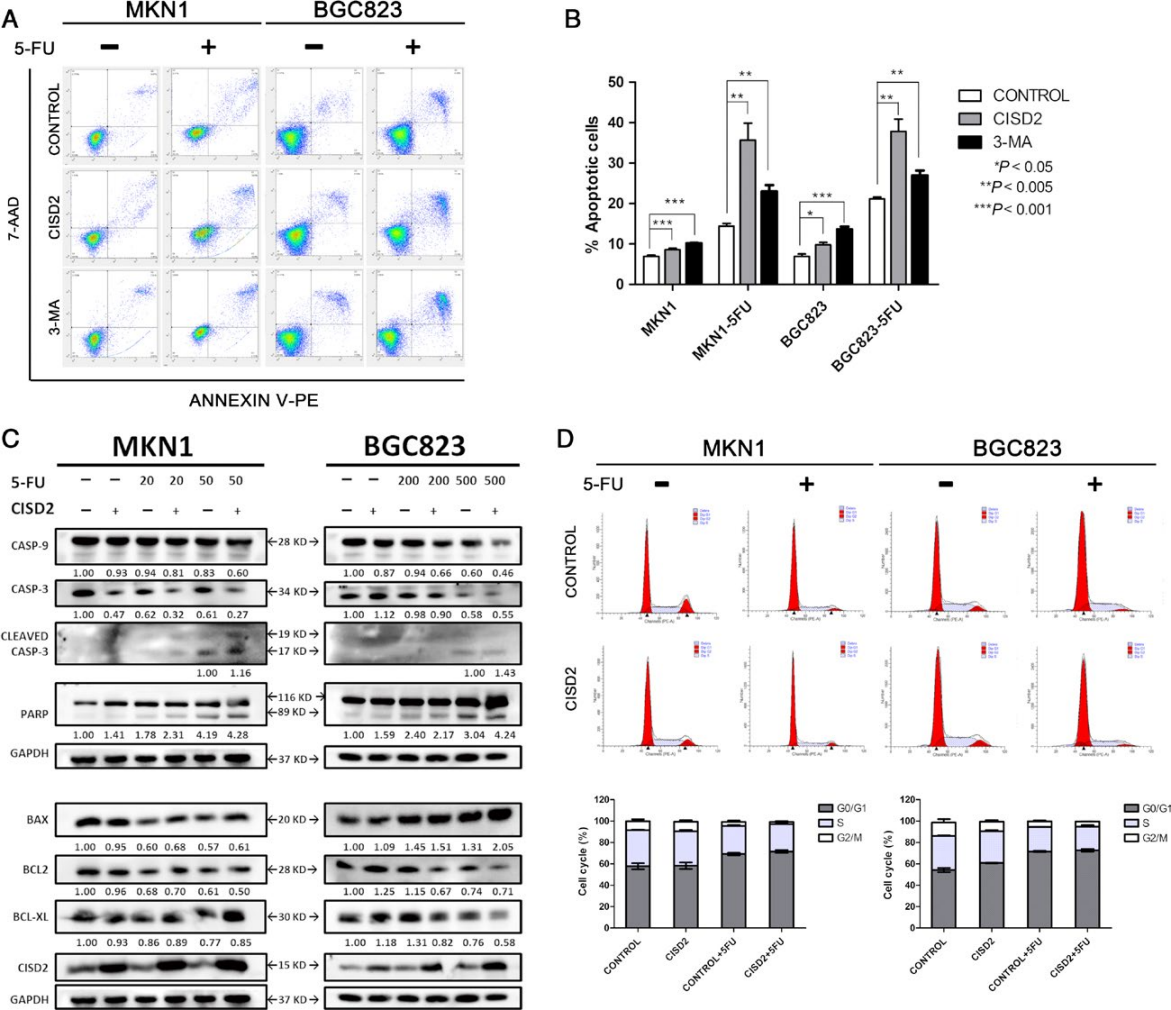
CISD2 is associated with 5‐FU induced apoptosis but not cell cycle arrest. (A and B) MKN1 and BGC823 cells were relatively divided into three groups: control, CISD2 and control+3‐MA, and apoptosis were analysis after 48 h cultured with or without 5‐FU treatment at the indicated dose. Image shows a representative experiment out of three. (C) CISD2 overexpression and control cells were treated with 5‐FU in MKN1 and BGC823 for 48 h. Total cell lysates were blotted against the indicated antibodies. GAPDH was used as loading control. (D and E) Cell cycle of MKN1 and BGC823 cells with or without 5‐FU treatment 48 h at the indicated dose was analyzed by flow cytometry. Image shows a representative experiment out of three. Data was performed as mean ± SD of three independent experiments. (*P <* *0.05, **P <* *0.005, ***P <* *0.001)

Tumor growth is often identified as a balance between proliferation and apoptosis 23. To examine the contribution of apoptosis to the suppression of growth by CISD2, cell apoptosis was assessed by double staining with Annexin V‐PE and 7‐AAD. The percentages of apoptotic cells in the CISD2 group were slightly higher for both MKN1 (8.59 ± 0.31% vs. 6.92 ± 0.28%, *P *= 0.0005) and BGC823 cells (9.78 ± 0.52% vs. 6.95 ± 0.45%, *P *= 0.0087) than those in control group (Fig. 5A). Furthermore, cells in the CISD2 overexpression group and control group were exposed to 5‐FU (100 *μ*mol/L for MKN1 and 1000 *μ*mol/L for BGC823 cells), and a significant increase was observed in the number of apoptotic MKN1 cells in the CISD2 group (35.64 ± 3.64% vs. 14.39 ± 0.572%, *P *= 0.0013) and BGC823 cells in the CISD2 group (37.80 ± 2.49% vs. 21.13 ± 0.37%, *P *= 0.0096) compared with cells in the control group (Fig. 5A and B). In keeping with this finding, the expression of apoptosis‐related proteins was detected by Western blotting. In 5‐FU treated cells, the following executioner caspases were cleaved into their specific active forms: caspase‐3, from 34 KD to 19 KD and 17 KD, and PARP, from 116 KD to 89 KD. The total amount of caspase‐9 was decreased in treated cells compared with untreated cells. Compared with control cells, cells with ectopic expression of CISD2 had significantly enhanced cleavage of caspase‐3 and PARP (Fig. 5C top panel). In addition, the BCL‐2 family proteins are known to be closely related to apoptosis. It was found that the level of the proapoptotic protein BAX was increased whereas the level of the antiapoptotic protein BCL‐2 was decreased in cells in the CISD2 group that were treated with 5‐FU compared with the control group. Interestingly, the anti‐apoptotic protein BCL‐xl decreased in BGC823 cells that overexpressed CISD2, but no significant change was seen in MKN1 cells compared with control cells (Fig. 5C bottom panel).

With respect to the cell cycle distribution, stable CISD2 overexpression had no significant effect on either MKN1 or BGC823 cells (Fig. 5D). After exposure to 5‐FU for 48 h, both cell lines exhibited a decrease in the percentage of cells in S phase accompanied by an increase in the percentage of cells in *G*0/*G*1 phase. Flow cytometric analysis showed that after treatment with 5‐FU, the percentage of cells in *G*0/*G*1 phase (69.17 ± 1.65%) was significantly higher than that in untreated MKN1 cells (59.05 ± 2.28%, *P *= 0.0414). In the CISD2 group, the data were 71.63 ±2.38% and 58.25 ± 4.29% respectively, (*P *= 0.0178). Similar results were obtained in BGC823 cells. Therefore, it was suggested that 5‐FU could repress cell cycle progression in GC cells. However, no significant change was seen in the cell cycle distribution when the CISD2 group was compared with the control group (Fig. 5D and E). Taken together, these results suggest that CISD2 is associated with sensitivity to 5‐FU through its enhancement of apoptosis rather than cell cycle arrest.

### 5‐FU treatment increases CISD2 expression in GC cells

**Figure 6:**
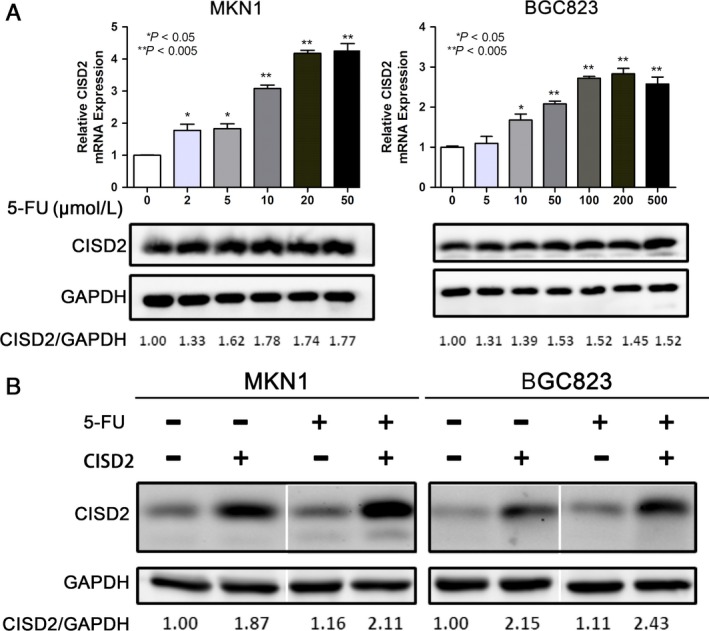
5‐FU treatment increases CISD2 expression in GC cells. (A) Effect of 5‐FU treatment on CISD2 expression level in MKN1 and BGC823 cells. MKN1 and BGC823 cells were treated with 0, 2, 5, 10, 20, 50 *μ*mol/L and 0, 5, 10, 50, 100, 200, 500 *μ*mol/L of 5‐FU respectively, and the expression of CISD2 protein was examined by qRT‐PCR and Western blotting 48 h later. (B) Effect of 5‐FU treatment on CISD2 expression level in stable CISD2 overexpression cells. CISD2 overexpression and control cells were treated with 50 *μ*mol/L and 500 *μ*mol/L of 5‐FU, respectively, and the expression of CISD2 was examined by Western blotting 48 h later. The values underneath the bands represent the densitometric estimation of the intensities of the bands.(*P <* *0.05, **P <* *0.005, ***P <* *0.001)

To further investigate the relationship between CISD2 and chemotherapeutic sensitivity of GC cells to 5‐FU, two parental cell lines were treated with different concentrations of 5‐FU. It was found that the expression of CISD2 was significantly increased after 5‐FU treatment in MKN1 and BGC823 cells (Fig. 6A). Furthermore, MKN1 and BGC823 cells with ectopic CISD2 expression and control cells were treated with 5‐FU for 48 h (50 *μ*mol/L and 500 *μ*mol/L for MKN1 and BGC823 cells, respectively), and a similar phenomenon was found that a significantly higher expression of CISD2 was observed in the CISD2 overexpression group after 5‐FU treatment (2.11‐fold for MKN1 and 2.43‐fold for BGC823 cells) (Fig. 6B). These results indicate that 5‐FU treatment could increase endogenous as well as exogenous overexpression of CISD2 in GC cells.

### CISD2 overexpression antagonizes 5‐FU‐induced autophagy via the activation of the AKT/mTOR pathway in GC cells

**Figure 7:**
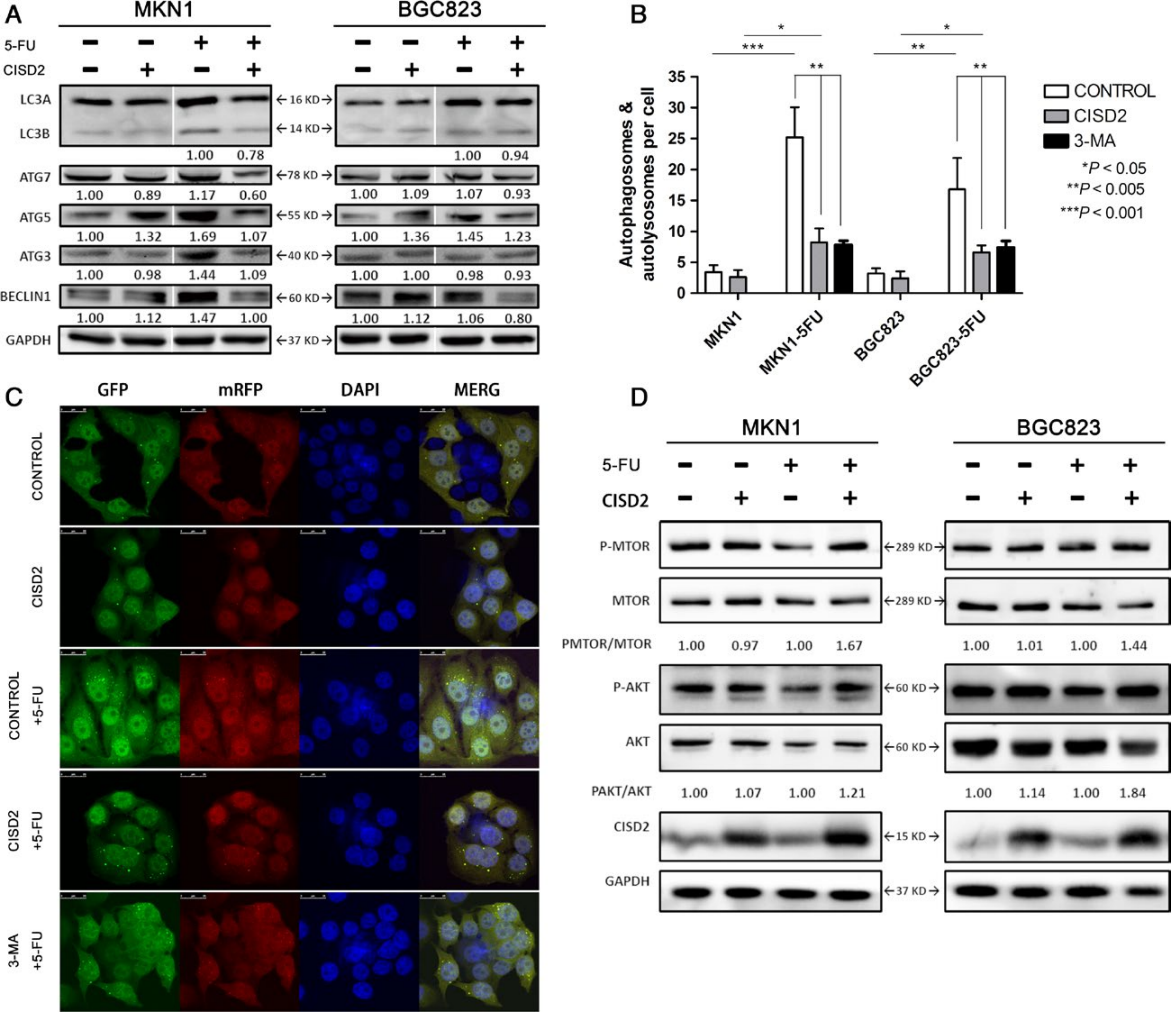
CISD2 overexpression antagonizes 5‐FU induced autophagy by activating the PI3K/AKT/mTOR pathway in GC cells. (A) LC3 and autophagy associated genes expression in GC cells with or without 5‐FU. Total cell lysates were blotted against the indicated antibodies. GAPDH was used as loading control. (B and C) Autophagosomes labeled by LC3 were observed in CISD2 overexpression, control and 3‐MA groups with or without 5‐FU in MKN1. Image shows a representative experiment out of three by a confocal microscope. Data are presented as mean ± SD of three independent experiments. (D) Effect of CISD2 under 5‐FU treatment on target in the AKT/mTOR signaling pathway. CISD2 overexpression and control cells were treated with 0, 50 *μ*mol/L in MKN1 and 0, 500 *μ*mol/L of 5‐FU in BGC823 for 48 h. Total cell lysates were blotted against the indicated antibodies. GAPDH was used as loading control. The values underneath the bands represent the densitometric estimation of the intensities of bands. (*P < 0.05, **P < 0.005, ***P < 0.001)

In the present study, we first verified that 3‐MA could inhibit 5‐FU‐induced autophagy (Fig. 7A and B) and increase 5‐FU‐induced apoptosis (Fig. 5A and B), which enhanced the susceptibility of GC cells to 5‐FU. Considering that CISD2 rendered GC cells more susceptible to 5‐FU, it was hypothesized that CISD2 could inhibit 5‐FU‐induced autophagy. During autophagy, cytosolic microtubule‐associated protein light chain 3A (LC3A) is converted into the membrane‐bound lapidated LC3B. When MKN1 and BGC823 cells were treated with 5‐FU for 48 h (50 *μ*mol/L and 500 *μ*mol/L for MKN1 and BGC823 cells, respectively), a higher ratio of LC3B/LC3A expression, and an increase in the protein level of Beclin1 and autophagy‐associated genes (ATG3, ATG5 and ATG7) were observed by Western blotting. However, the CISD2 group showed a lower ratio of LC3B/LC3A in response to 5‐FU, which is consistent with a remarkable down‐regulation of Beclin1 and ATGs compared with the control group after 5‐FU treatment (Fig. 7A).

Next, to assess the induction of autophagy after 5‐FU treatment, lentivirus labeled with mRFP‐LC3‐GFP, as described in the methods section, was stably transfected into two GC cell lines, and autophagosomes labeled by LC3 were observed by confocal fluorescence microscopy. As shown in Fig. 7B, the untreated cells presented diffuse LC3 staining or only small dots, while the 5‐FU‐treated cells presented a strong accumulation of autophagosomes. However, the treated cells exhibited a weaker accumulation when they were pretreated with 3‐MA, which indicated that 5‐FU could induce autophagy, as the autophagy inhibitor 3‐MA could reduce the induction of autophagy. Moreover, an apparent decrease in the accumulation of autophagosomes in the CISD2 group compared with the control group after 5‐FU treatment was found, which showed that CISD2 could reduce 5‐FU‐induced autophagy in both MKN1 and BGC823 cells. The merged images showed that the GFP and mRFP fluorescence was diffuse in untreated cells, while the fluorescence was more frequently punctate in 5‐FU‐treated cells in the control group (3.4 dots/cell vs. 25.2 dots/cell, *P *= 0.0005) and in the CISD2 group (2.6 dots/cell vs. 8.2 dots/cell, *P *= 0.0148). Compared with cells in the 5‐FU treated groups, the CISD2 group showed a remarkably attenuated number of autophagosomes in MKN1 cells (8.2 dots/cell and 25.2 dots/cell in MKN1 cells, *P *= 0.0042) (Fig. 7C) and BGC823 cells. The PI3K/Akt/mTOR pathway is the key regulator of autophagy. In a study of the molecular mechanism, we found that CISD2 effectively increased the phosphorylation levels of AKT (S473) and mTOR (Ser2448), and particularly, CISD2 reversed the decrease in p‐MTOR and p‐AKT induced by 5‐FU (Fig. 7D). These results suggest that ectopic CISD2 overexpression could antagonize 5‐FU‐induced autophagy via the activation of the AKT/mTOR pathway in both MKN1 and BGC823 cells.

## Discussion

Gastric cancer, which is the most common upper gastrointestinal tumor, has a high morbidity and mortality worldwide. 5‐FU‐based chemotherapy is the standard treatment course and is widely used in advanced gastric cancer. Considering the rapid development of 5‐FU resistance, the identification of novel markers that could predict response to chemotherapy, and the development of novel chemosensitizing strategies will lead to effective targeted strategies to combat GC. CISD2 is the second member of the protein family that contains the CDGSH iron sulfur domain and is localized in the mitochondrial outer membrane 11. Actually, CISD2 is an evolutionarily conserved gene that contains a transmembrane domain, a CDGSH domain and a conserved amino acid sequence for iron binding 24. Chang et al. identified CISD2 as a BCL‐2‐associated co‐factor that contributes to an enhanced BCL‐2‐Beclin1 interaction, which functionally antagonizes Beclin1 mediated autophagy 25. Through a structure‐function analysis 10, peptide array and DXMS analysis 26, Sagi Tamir et al. further revealed that CISD2 bind to small segments of the BH3 regions of BCL‐2. These findings indicated CISD2 as a BCL‐2 binding partner at a branch point between autophagy and apoptosis.

In this manuscript, we aimed to reveal the role of CISD2 in tumor development, metastasis and modulation of the sensitivity of GC cells to 5‐FU. The CISD2 expression profile was first examined in 52 pairs of GC samples by mRNA analysis and in 197 pairs of human GC tissues by IHC. It was found that CISD2 was frequently down‐regulated in GC patient samples compared with adjacent normal tissues, which combined with data from a public database showed frequent copy number loss of *CISD2* in human gastric cancer. A subsequent clinicopathological analysis indicated that CISD2 was significantly correlated with some parameters including age, Lauren's classification, and differentiation, but no significant correlation was observed in terms of postoperative survival. Based on the mRNA and protein expression levels in GC cell lines, CISD2 overexpression models were constructed using lentiviral infection. The results of the cell function assay demonstrated that CISD2 could inhibit GC cell proliferation and metastasis and that CISD2 could slightly increase apoptosis. Exposure of GC cells to different concentrations of 5‐FU ‐suggested that CISD2 expression was elevated in a dose‐dependent manner in GC cell lines. Furthermore, it showed that CISD2 could dramatically reduce the IC50 value of 5‐FU of MKN1 and BGC823 cells. Therefore, we propose that CISD2 may be closely associated with chemosensitivity in GC, and we have attempted to clarify the mechanism of increased chemotherapy sensitivity.

For several decades, apoptosis has been considered the elementary mechanism of programmed cell death in mammalian cells 27. However, accumulating evidence suggests that the validity of anticancer therapies is not confined to apoptosis but that it also involves autophagy. Some chemotherapeutic drugs including 5‐FU can induce protective autophagy, and thus the blockade of cancer cell autophagy is regarded as a novel approach to improve the efficiency of chemotherapy in cancer treatment 28, 29, 30. In the present study, it was first verified that 5‐FU could induce apoptosis as well as autophagy in MKN1 and BGC823 cells. When the cells were pretreated with the autophagy inhibitor 3‐MA, the increased number of apoptotic cells and the attenuation of the accumulation of autophagosomes in GC cells verified that autophagy had a protective effect on 5‐FU cytotoxicity. Therefore, antagonism of 5‐FU‐induced protective autophagy helps to enhance the chemotherapeutic sensitivity of GC cells.

The BCL‐2 protein family regulates and contributes to programmed cell death in the mitochondria 31. Additionally, CISD2 was found to be displaced from BCL‐2 by BIK, which is a member of the BH3‐only protein family; this resulted in the release of Beclin1 from BCL‐2 inhibition 10. In this manuscript, we showed that ectopic CISD2 overexpression could significantly increase apoptosis after 5‐FU treatment through a caspase cascade in MKN1 and BGC823 cells. We also observed that the level of BAX was increased while that of BCL‐2 was decreased as a result of 5‐FU treatment in both MKN1 and BGC823 cells. Thus, CISD2 could enhance the susceptibility of GC cells to 5‐FU via an increase in 5‐FU‐induced apoptosis through the mitochondrial‐mediated caspase cascade. Furthermore, the BCL‐2 family members share one or more of the four characteristic BH domains, whereas Beclin1 is a nonmembrane protein and has a weak BH3 domain 32. When the BH3 region is occupied due to the ectopic overexpression of the CISD2 protein, BCL‐2 does not anchor the Beclin‐1 complex to the endoplasmic reticulum (ER) and thus autophagy is inhibited. Therefore, CISD2 overexpression could inhibit autophagy under 5‐FU treatment conditions. In this study, two groups of cells were treated with 5‐FU for 48 h. The lower ratio of LC3B/LC3A and the down‐regulated Beclin1 and ATG expression were consistent with a decreased accumulation of autophagosomes, which was detected in the CISD2 overexpression group. The data showed that CISD2 could recede 5‐FU‐induced protective autophagy to increase the susceptibility of GC cells to 5‐FU.

Convincing evidence shows that the PI3K/Akt/mTOR pathway represents the major negative regulator of autophagy 33. AKT activation leads to activation of downstream mTOR, which is a critical factor for autophagy regulation, as it plays a role in the antagonism of autophagy 34. In this study, it was suggested that CISD2 activated the AKT/mTOR pathway and that the decrease in AKT phosphorylation induced by 5‐FU returned in cells with ectopic CISD2 overexpression. Thus, activation of the AKT/mTOR pathway by CISD2 could inhibit 5‐FU‐induced autophagy. Chemotherapy drugs can also induce autophagy at an early disease stage as a survival mechanism, which may contribute to resistance to apoptotic death 35. Thus, the modulation of autophagy represents a promising approach to enhance the potency of apoptosis. In addition, the selective regulation of AKT/mTOR signaling, such as screening for CISD2 expression, might prove valid for cancer treatment when combined with 5‐FU.

This study shows that CISD2 is down‐regulated in gastric cancer tissues and cells, and its effects on the inhibition of gastric cancer cell proliferation, migration, invasion, and the enhancement of chemotherapy sensitivity are mediated by the increase in 5‐FU‐induced apoptosis and the inhibition of autophagy through the AKT/mTOR pathway. The results demonstrate a potential mechanism that underlies the tumor‐suppressor role of CISD2, and it is expected that screening for CISD2 expression combined with chemotherapy drugs will become a new therapeutic strategy for gastric cancer.

## Conflict of Interest

The authors have read the journal's policy on the disclosure of potential conflicts of interest and have none to declare.
